# ‘An eye-opener and helpful intervention strategy’: Experiences and perceptions of community members on the volunteer-led community strategy on lung cancer awareness and mobilisation in KwaZulu-Natal

**DOI:** 10.4102/jphia.v17i1.1609

**Published:** 2026-10-08

**Authors:** Sthabile W. Mtolo, Themba G. Ginindza, Siyabonga B. Dlamini

**Affiliations:** 1 Discipline of Public Health, College of Health Sciences, University of KwaZulu-Natal, Durban, South Africa; 2 Cancer and Infectious Diseases Epidemiology Research Unit, College of Health Sciences, University of KwaZulu-Natal, Durban, South Africa

**Keywords:** lung cancer, awareness, mobilisation, community health workers, volunteer-led intervention

## Abstract

**Background:**

Community health awareness initiatives are important for promoting health, encouraging early prevention and improving health-seeking behaviour. Lung cancer remains the leading cause of cancer-related mortality globally.

**Aim:**

This study explored community members’ experiences and perceptions of a volunteer-led lung cancer awareness and mobilisation strategy.

**Setting:**

The study was conducted in KwaZulu-Natal, South Africa, across Durban (Umlazi, Chatsworth, and South Durban Basin) and Pietermaritzburg (Imbali and Sobantu).

**Methods:**

A qualitative design was used involving 13 adult community members who had received lung cancer education through a volunteer-led intervention implemented by the Cancer Association of South Africa-Multinational Lung Cancer Control Programme. Focus group discussions explored participants’ experiences of the intervention. Participants were selected based on availability, age (18 years and older) and prior exposure to the programme. Data were analysed using Braun and Clarke’s thematic analysis.

**Results:**

Participants recognised the value of the volunteer-led strategy and reported positive impacts on health knowledge, health-seeking behaviour, community engagement and cultural receptivity. Suggestions for improvement included greater community involvement, stronger cultural integration and enhanced feedback mechanisms. Overall, participants viewed the initiative as an effective approach for increasing community awareness and education about lung cancer.

**Conclusion:**

Community health volunteers are increasingly recognised as important contributors to health promotion. Findings highlight the need for culturally grounded lung cancer awareness strategies and supportive health policies.

**Contribution:**

To inform policy development on community-level lung cancer awareness and mobilisation, with a focus on understanding community members’ experiences of these approaches, particularly within underserved populations.

## Introduction

Community health awareness and screening for lung cancer are important variables in eradicating the increasing mortality rates of lung cancer. In the United States (US), the average 5-year survival rate of lung cancer is a low 20%.^1^ However, when diagnosed early, the survival rate is higher at approximately 50%.^1^ Lung cancer screening could save lives if annual screening with low-dose computed tomography (LDCT) was performed to ensure early detection and treatment.^2^ The uptake of LDCT for lung cancer screening is often extremely low, and therefore, the need for screening initiatives, especially among disparate groups and low socioeconomic status groups continues to rise.^3^ Niranjan and colleagues argue that the utilisation of community health advisors to encourage awareness and screenings is an essential strategy to reduce the lung cancer burden.^4^ In a study on knowledge, attitudes and practices towards lung cancer in KwaZulu-Natal (KZN), many participants shared that they had never screened for lung cancer.^5^ Lung cancer awareness often increases knowledge about lung cancer signs and symptoms, and risk factors, and motivates screening for early diagnosis and treatment.^6^ Better awareness and screening for lung cancer are essential.

The health system plays a significant role in incorporating community health workers (CHWs) into the healthcare system. Community healthcare workers are well known to bridge the gap between individuals and health facilities.^7^ In their study, Rachlis and colleagues found that CHWs are usually accepted in the communities they serve, mainly because of their essential role in improving knowledge, promoting health behaviour change and effectively contributing to serving the community with proper disease management.^7^ Limited health awareness and preconceived fears may affect positive health-seeking behaviour.^7^ Harris and colleagues describe delays in health-seeking behaviour as contributing to increased mortality rates of lung cancer.^8^ This study aimed to explore the experiences and perceptions of ‘users’ (community members) regarding the volunteer-led community strategy on lung cancer awareness and mobilisation.

## Research methods and design

### Study design

A qualitative study was conducted among community members who were educated on lung cancer as part of the collaborative initiative between Cancer Association of South Africa (CANSA) and Multinational Lung Cancer Control Programme (MLCCP). The study used an interpretive qualitative design, as this approach is appropriate when the aim is to explore participants’ experiences within a specific social context.^9^ Using this design, we sought to understand the users’ experiences of the lung cancer awareness intervention and how they made meaning of those experiences. This study, therefore, used focus group discussions (FGDs) to enhance conversations about the community members’ perceptions and experiences.

### Study setting

The study was conducted in partnership with the Cancer and Infectious Diseases Epidemiology Research Unit, University of KwaZulu-Natal. This study formed part of a larger MLCCP project, which has been ongoing for more than 2 years, in different phases, and focuses on educating the public about lung cancer. As part of the intervention, 20–30 community volunteers were identified, trained on lung cancer awareness and received a monthly stipend to conduct door-to-door awareness, as well as community and clinic-based events. For this specific study, only individuals who received door-to-door education were included. The educational content covered the nature of lung cancer, its risk factors, signs and symptoms, and guidance on seeking healthcare. It was implemented through a volunteer-led programme, with trained community volunteers delivering the education to community members in the study sites via door-to-door visits, community gatherings and clinic presentations. Each door-to-door session lasted approximately 30 min – 40 min.The study was conducted in the KZN province of South Africa. Two metropolitan cities in the province were included, namely, Durban and Pietermaritzburg (PMB). Only these two municipalities were included in the broader MLCCP study, having been identified during the project’s initiation phase as the areas affected by lung cancer. The Durban sites were made up of Umlazi, Chatsworth and the South Durban Basin, the latter consisting of Lamontville, the Bluff and Wentworth. The PMB sites were Imbali and Sobantu.

### Study population and sampling strategy

As part of the larger study, the community volunteers had been equipped with the necessary skills for lung cancer education and therefore played an important role in educating their communities. The community volunteers were males and females, adults older than 18 years, the majority were African people, isiZulu speaking, others were mixed race and English speaking. Some of the volunteers were already under the Department of Health as CHWs.

The researcher accessed the lung cancer awareness database of community members educated by volunteers at different sites, which had over 6000 records and about 11 400 community members educated, to identify and extract their contact details. The database was on REDCap, a prospective data collection software used by CANSA MLCCP for the monitoring and evaluation of project implementation. A total of 40 community members with valid contact numbers were contacted and invited to participate in the FGDs. The purposive sampling technique was used to determine the 40 community members contacted, particularly the maximum variation sampling to obtain diverse views from community members targeted for this study.^10^ This sampling strategy is optimal for sourcing diverse views from a variety of participants who are deemed to be information-rich on the topic of interest. Thirteen community members were available to participate, making the sample size of the study. The sampling of the participants ensured that each study site was accounted for, so that every community in the study had at least two participants.

The participants’ criteria to participate in the study included:

- Age group, 18 years and older.
- Must have learned about lung cancer from volunteers.
- Captured on Redcap – lung cancer awareness database.

### Data collection

To encourage discussions among the community members on their experiences, two FGDs were conducted. The FGDs were held in two venues: (1) a community hall and (2) a local hospital. The volunteers were transported to these venues by the research team. The FGDs allow probing and follow-up questions to explore further what is being discussed.^11^ This encourages full expressions of experiences lived, to gather rich data, which was also carried out to guarantee study triangulation. The data collection process for the MMedSci study as a whole spanned from April 2023 to November 2023, with the FGDs for this objective (community members’ experiences-users) conducted in October 2023. The community members were exposed to the intervention between 3 months and 6 months prior to the conduct of the FGDs. Two research assistants with prior knowledge of lung cancer awareness and experience in qualitative research methodologies assisted with the data collection, including taking notes during these FGDs, while the researcher facilitated the FGDs.

The topics explored during the FGDs included the community members’ understanding of the intervention strategy, their perceptions and their lung cancer knowledge post the intervention strategy. Community acceptance, health behaviour and attitude change as a result of lung cancer insight and awareness were covered. Possible future improvements of such intervention strategies were explored. The local language used during the FGDs was isiZulu. Permission was sought from all participants to record the discussions. To maintain anonymity, each participant was assigned a number, which was used to indicate their responses in the transcripts rather than their name.

### Data analysis

The translated transcripts were analysed using the thematic analysis steps as indicated by Maguire and Delahunt.^12^ The authors state that analysing data thematically entails using identified patterns in the data collected and forming them into themes that interest or cover certain issues and topics closely related to the research study. The researchers followed the steps for thematic analysis, where we familiarised ourselves with the data by constantly listening to the recordings and reading the transcripts. This enabled us to work on the data by engaging with it and understanding the patterns. While immersing with the data, we assigned codes and formed them into themes. Lastly, elaborating, interpreting and thoroughly checking the analysed data. The NVivo 14-2023 software (Lumivero, Denver, Colorado State, United States [US]) organised and classified data according to the study research questions and objectives. Verbatims have been used to depict participants’ responses.

### Measures to ensure trustworthiness

Trustworthiness was established through several strategies aligned with qualitative research standards.^13^ The study used audio recordings, with the participants’ permission, and note-taking to make sure that the researcher received the information from participants as it was being shared, ensuring the credibility of the study. The recorded discussions were later transcribed and translated from isiZulu to English. Dependability was maintained through consistent documentation of the research process and the use of verbatim quotations to support the findings. The study’s transferability was achieved by having substantial descriptions of the research findings; this is sufficient information about the participants, context, results and the research process. Confirmability was supported by a clear audit trail, including audio recordings, transcripts and field notes, as well as the use of thematic analysis to guide data interpretation and mitigate researcher bias. Reflexivity was therefore maintained through continuous critical reflection on my positionality, assumptions and potential influence on both data collection and analysis. This included maintaining a reflexive journal to document methodological decisions, personal reflections and emerging biases, thereby enhancing transparency and ensuring that the findings remained grounded in participants’ lived experiences rather than my own preconceptions.

### Ethical considerations

This study obtained ethics approval from the University of KwaZulu-Natal Biomedical Research Ethics Committee (BREC) (reference no. BREC/00005350/2023) and gatekeeper permission from CANSA. The informed consent process was explained and discussed, and community members were encouraged to read the study information. They were informed of the voluntary nature of their participation. Each participant read the information sheet individually. Where needed, the researcher addressed any questions, and participants signed the informed consent form individually.

## Results

The FGDs involved a total of 13 community members (Table 1). As mentioned above, participants came from the identified communities of the study, Durban and PMB. The PMB FGD had 5 participants, and the Durban FGD had 8 participants. They comprised 11 females and 2 males, aged between 21 years and 63 years. The community members’ lung cancer awareness database has mostly African people participants. Therefore, all 13 participants were African people and isiZulu speaking.

**TABLE 1:** The profile of community members who participated in the focus group discussion about the intervention on lung cancer awareness and mobilisation.

Participant no.	Gender	Age (years)	Ethnicity	Level of education	Site
1	Male	32	African person	Secondary	PMB-Imbali
2	Female	32	African person	Secondary	PMB-Imbali
3	Female	39	African person	Secondary	PMB-Imbali
4	Female	26	African person	Secondary	PMB-Imbali
5	Female	26	African person	Secondary	PMB-Sobantu
6	Female	24	African person	Tertiary	Umlazi
7	Female	63	African person	Tertiary	Umlazi
8	Female	26	African person	Secondary	Chatsworth
9	Female	30	African person	Secondary	Chatsworth
10	Female	24	African person	Secondary	Chatsworth
11	Female	30	African person	Secondary	Umlazi
12	Female	21	African person	Secondary	Chatsworth
13	Male	40	African person	Secondary	Umlazi

PMB, Pietermaritzburg.

The results are presented in main themes and subthemes in Table 2.

**TABLE 2:** Results by main themes and subthemes.

Themes	Subthemes
1. Health awareness and behaviour change through a volunteer-led intervention	1.1. Increased health awareness 1.2. Behavioural change
2. Volunteer-led strategy educational intervention methods	2.1. Home visits – ‘A personal touch’ 2.2. Community gatherings
3. Barriers to effective health education in volunteer-led interventions	3.1. The influence of culture on health education 3.2. Communication and acceptance of health education
4. Ideas on programme improvement	4.1. Increased community involvement 4.2. Cultural integration 4.3. The importance of feedback

### Theme 1: Health awareness and behaviour change through a volunteer-led intervention

The first theme discusses lung cancer awareness and its impact at the community level. It also discusses behavioural changes that have occurred during or after the intervention. This theme dives into participant feedback regarding their degree of knowledge following lung cancer education, and how this affected their behaviour. Increased health awareness and behavioural change are the two subthemes within this theme.

#### Subtheme 1.1: Increased health awareness

An increase in health awareness was evident among participants, who reported a heightened understanding of lung cancer, particularly in relation to its symptoms and severity. Participants from Chatsworth demonstrated greater awareness of what signs and symptoms to look out for, reflecting an improved ability to recognise potential indicators of the disease. A 26-year-old female participant highlighted learning about the broader implications of common symptoms, which changed their understanding and awareness of lung cancer. She stated:

‘There is something I learned that coughing is flu, but when a person has been coughing a lot, that is TB symptoms. I did not know that coughing a lot can also be a symptom of lung cancer.’ (P8, Female, 26 years, Durban-Chatsworth)

Participants, both males and females, from the Umlazi and PMB sites, reported that the educational sessions encouraged a proactive approach to their health. This indicates the intervention’s effectiveness in increasing lung cancer awareness and influencing personal health practices. Participants also described changes in health-risk behaviours, particularly smoking, which they attributed to the targeted health education provided. These changes demonstrate the intervention’s broader impact on lifestyle choices associated with lung cancer risk. One of the participants stated that:

‘It was very helpful even for some of my bad habits, like when I go to places for entertainment, where when I drink, I would feel like having a smoke, I had to stop that habit since I was warned about smoking and other things.’ (P2, Female, 32 years, PMB-Imbali)

The strategy effectively instilled a sense of urgency and importance around regular health checks. This led to behavioural changes such as frequent clinic visits for symptoms previously dismissed as minor ailments. Participants described a deep sense of vigilance towards health symptoms such as coughing, which they often dismissed previously as trivial, as elaborated by P2:

‘When he told me about coughing yoh! Even now I don’t take coughing lightly, I just go to the clinic. Even with the neighbours, I tell them to go to the clinic. It was very helpful, even for some of my bad habits.’ (P2, Female, 32 years, PMB-Imbali)

#### Subtheme 1.2: Behavioural change

Community members from the PMB-Imbali site described a significant change in how they respond to potential symptoms of lung cancer, such as persistent coughing, showing an immediate and severe approach to health checks. Participant 4 (P4) shares their reaction to some of the lung cancer symptoms learned from the volunteers during lung cancer education:

‘They said if you cough blood or unending coughing, for me, two days is enough, I go straight to the clinic, as far as it is, but it seems as if it is very close when I notice symptom-like coughs.’ (P4, Female, 26 years, PMB-Imbali)

All the participants acknowledged and agreed with one another that the intervention strategy brought awareness to the community, which led to transformative action. For instance, PMB participants shared that it has ushered in and led most community members to adopt an early intervention approach to health. They highlighted how the strategy effectively motivated the community to seek early medical evaluations, potentially preventing late-stage disease diagnosis:

‘It did help a lot. The door-to-door helped people go check because other people wouldn’t think the sickness would affect them up to the stages where you go check, and it’s already late; a lot of them in my community got help from door-to-door.’ (P1, Male, 32 years, PMB-Imbali)

Still on the subtheme of behavioural change, P13 below goes on to share how the intervention encouraged them to take proactive health practices:

‘I am a male who drinks and smokes; in 2016 or 2015, I had TB and was able to survive; I finished treatment, and still, I went back to drinking and smoking. Until they came home, especially we male people when such people come, we find a way to run. However, I listened and decided to make health changes in my drinking and smoking habits.’ (P13, Male, 40 years, Durban-Umlazi)

This quote reflects a transformation in the participants’ perception and behaviour towards health, especially after surviving tuberculosis and interacting with the lung cancer awareness campaign.

Participants from PMB-Imbali and Durban-Umlazi sites discussed the tangible change in family behaviours concerning smoking. This was directly influenced by the awareness raised through the education intervention. This illustrates the impact of the lung cancer awareness strategy on individual and community health behaviours. They help demonstrate the volunteer-led intervention strategy’s effectiveness in enhancing disease awareness and motivating proactive health practices, as described by P2:

‘My father used to smoke in front of us and we were not bothered by that but after TZ [*volunteer*] came saying the people who get sick the most are us because when he puffs … the smoke comes to us, we are the ones who get hurt so from then we told him to smoke outside.’ (P2, Female, 32 years, PMB-Imbali)

### Theme 2: Volunteer-led strategy educational intervention methods

This theme encompasses various strategies to involve the community and impart knowledge about health matters actively. These strategies range from personalised approaches, such as visiting individual households, to more inclusive ones, like organising community meetings or workshops. The efficacy of these strategies may differ depending on several aspects, such as community preferences, cultural norms and the presence of community members. This theme is essential for understanding the effectiveness of different outreach techniques in raising awareness, imparting crucial health information and encouraging community involvement in health issues.

#### Subtheme 2.1: Home visits – ‘A personal touch’

Participants from Durban and PMB sites reflected on the strengths and limitations of different educational methods. Sharing more about the personal touch of home visits, which they appreciated, as elaborated by P10 and P2 below. Most participants felt that the home visit demonstrated care and concern, thus making the information more relatable and impactful:

‘When they visit you at home, you feel they care about us here in the community, it makes you listen more. There’s something about someone taking the time to come to your door that makes you pay attention. It’s like they’re saying, “This is important, and so are you.”’ (P10, Female, 24 years, Durban-Chatsworth)

‘It’s much more personal and direct. You can ask questions in the privacy of your own home, and it feels safer to discuss personal issues that you might not want to talk about in a public meeting.’ (P2, Female, 32 years, PMB-Imbali)

In addition, participants from the PMB Sobantu site acknowledged the value of door-to-door outreach. They indicated its ability to reach individuals who might not attend larger community meetings, thereby ensuring comprehensive information dissemination. Participant 5 (P5) elaborates:

‘Door-to-door is good because they catch people who normally don’t come out to meetings. Some people are shy or they’re just not the type to go to big gatherings, especially the older ones or those who are busy. This way, everyone gets the information they need right at their doorstep. It ensures that no one is left out because they’re afraid to ask questions in a big group or because they couldn’t make it to the community centre.’ (P5, Female, 26 years, PMB Sobantu)

#### Subtheme 2.2: Community gatherings

Pietermaritzburg Imbali and Durban Chatsworth sites participants suggested that community meetings could be more effective in ensuring that more individuals can receive information. They suggested that while door-to-door visits ensure personalised interaction, group meetings in communal spaces such as halls could accommodate larger audiences at once, facilitating a more efficient educational process:

‘They should maybe have meetings in halls to explain these because not everyone is free when they come to the houses.’ (P1, Male, 32 years, PMB Imbali)

Participants such as P12 suggest that this method also facilitates ongoing community dialogue about health topics, as individuals can continue discussions outside the formal gathering based on a shared foundation of knowledge:

‘I think it’s better when they gather us at the community centre because we can all hear the same thing and ask questions. It helps when we are all together; it feels more like a community effort, and you can see that everyone is learning the same thing at the same time. It makes it easier to discuss it later with friends and neighbours because we all received the same message.’ (P12, Female, 21 years, Durban-Chatsworth)

Participants from PMB Imbali and PMB Sobantu sites appreciated interactive workshops incorporating visuals and narratives to educate on lung cancer. They stressed that the method facilitated a deeper understanding and retention of information by actively involving community members in the learning. Participant 4 (P4) below shared her experience of one of the workshops she attended:

‘We had a workshop where they used visuals and real-life stories. It made it so much clearer why we need to be aware of lung cancer signs. This interactive approach kept everyone engaged and allowed for immediate feedback.’ (P4, Female, 26 years, PMB-Imbali)

### Theme 3: Barriers to effective health education in volunteer-led interventions

#### Subtheme 3.1: The influence of culture on health education

Cultural receptivity and barriers explore how cultural factors influence the reception of health information within communities. It looks at the cultural norms and beliefs that the community members thought hindered the effective communication and the implementation of the health initiative in the volunteer-led lung cancer awareness strategy. Participants in both Durban and PMB sites shared that some cultural factors played an important role in how the information was received. The stigma around the illness was explained as discussing diseases such as cancer can cause them to manifest, so such conversations are usually ignored:

‘People here sometimes fear that talking about cancer can bring it into their lives like speaking it makes it real. So, they avoid the topic altogether.’ (P3, Female, 39 years, PMB-Imbali)

‘In our culture, talking about something like cancer is sometimes seen as a bad omen. It’s difficult for people to come out and discuss it openly without feeling that they are inviting trouble.’ (P7, Female, 63 years, Durban-Umlazi)

Some community members emphasised the impact of traditional beliefs on their ability to effectively engage in the intervention and the health conversation. They expressed a preference for traditional medicine over modern medical practices among some community members. This was seen mostly among the older population. Such beliefs were said to have challenged Western medical health education implementation. Participant 7 (P7) explains that:

‘Many older people still believe in traditional medicine and think that going to the hospital is a last resort, which delays many from getting early diagnosis and treatment.’ (P7, Female, 63 years, Durban-Umlazi)

A few participants from the older age group, such as P13, shared that there is normally resistance to modern healthcare that hinders the effectiveness of such health interventions:

‘Older people often dismiss what doctors say, preferring prayers and traditional healers over hospital treatments. They say it’s more aligned with our way of life.’ (P13, Male, 40 years, Durban-Umlazi)

#### Subtheme 3.2: Communication and acceptance of health education

A key point highlighted by participants was the importance of effective communication in health campaigns. Participants such as P11 from Durban, Umlazi site, observed that healthcare providers should prioritise linguistic inclusivity to make messages accessible and culturally appropriate. Failing to deliver health education in the local language creates a linguistic barrier, potentially disadvantaging the intended recipients:

‘The way health workers talk, using so much medical jargon, it’s like they speak another language. We need this in Zulu, something we can understand.’ (P11, Female, 30 years, Durban-Umlazi)

Participants such as P4 and P2, both from PMB sites, expressed a considerable reluctance to accept new information, specifically suspicion in the community towards outsiders with new health information. They reflected on common scepticism towards health initiatives led by outsiders, perceived as having ulterior motives. Such mistrust can be seen as a significant barrier to the acceptance and success of health programmes in specific communities:

‘There’s a lot of mistrust when new people come into our area talking about diseases. People wonder why they are here and if their intentions are good.’ (P4, Female, 26 years, PMB-Imbali)

‘There’s a suspicion about why these programmes target us; some think it’s a way to experiment on us rather than help us.’ (P2, Female, 32 years, PMB-Imbali)

Gender dynamics were identified as a concern in health education. Female participants expressed the gender-related cultural norms that affect how health education sessions are perceived and participated in within the community. A female participant, P7, described that:

‘In our meetings, it’s tough for women to speak about personal health issues. There’s a lot of shame and shyness because of our cultural norms.’ (P7, Female, 63 years, Durban-Umlazi)

Participant 9 (P9) explained the gender-specific barriers that affect women, highlighting how cultural norms can restrict open discussion among women about health issues in public forums:

‘Sometimes, it’s hard for women, especially younger ones, to speak up about their health in front of men, which makes community meetings challenging.’ (P9, Female, 30 years, Durban-Chatsworth)

### Theme 4: Ideas on programme improvement

#### Subtheme 4.1: Increased community involvement

This theme captures the voices of the community, and what they think could be done better or differently to make health interventions more effective. This is crucial as it leverages the collective experience and insights of individuals directly impacted by the efforts, responding to the grassroots level on what is effective and what may be enhanced. It encompasses everything from the content and delivery of health messages to the frequency and engagement methods. Some suggestions included adjusting the methods of delivering health education, such as incorporating more culturally relevant materials, using language and terminology that are easier for the community to understand or employing more engaging and interactive educational tools.

Community members suggested the adoption of more accessible communication strategies, such as the use of visual aids, simpler language and modern technologies like SMS and mobile apps. These tools are perceived as ways to make information more understandable and to keep the community engaged and informed on an ongoing basis. They stressed the need for visual aids to help overcome literacy barriers and make information more accessible and understandable. Participant 1 (P1) and P2 suggested as follows:

‘If they could use more visuals, like charts or videos, it would make it much clearer for everyone, especially for those who can’t read well.’ (P1, Male, 32 years, PMB-Imbali)

‘Using SMS to send reminders or health tips could help people remember to take their medications or follow up with their doctors.’ (P2, Female, 32 years, PMB-Imbali)

This recommendation highlights the potential of integrating technology to maintain a continuous engagement with the community, facilitating better health practices and adherence to treatment protocols.

Many participants emphasised using local resources and personnel, such as community leaders and respected figures, to enhance the credibility and acceptance of the health messages. They believe that messages delivered by familiar faces would be more warmly received and more likely to be trusted:

‘Involving local leaders more in the health talks could make a big difference. They are trusted and can influence many people to take the messages seriously.’ (P9, Female, 30 years, Durban-Chatsworth)

Finally, participants such as P10 suggested engaging community members in the creation and execution of health programmes to ensure that the initiatives are tailored to the community’s specific needs and concerns, fostering greater involvement and ownership. Moreover, many participants desired active participation in planning and implementing health programmes:

‘We should have more community-driven initiatives where we, the community members, help plan and execute the programmes. That way, it feels more relevant to us.’ (P10, Female, 24 years, Durban-Chatsworth)

#### Subtheme 4.2: Cultural integration

Another prominent suggestion in the participants’ responses was the need for cultural integration. They stressed the role of cultural beliefs in health practices, and suggestions were made for integrating traditional and modern healthcare practices through workshops or educational sessions. According to the participants, incorporating traditional healers could bridge the gap between modern and traditional medicine, enhancing community trust and receptivity to health programmes. Participant 13 (P13) suggested that:

‘Health programmes need to respect our traditions and maybe even include some of our local healers in their sessions.’ (P13, Male, 40 years, Durban-Umlazi)

Some participants emphasised that adapting health messages to fit the cultural and linguistic landscape of the community could also work:

‘Messages that reflect our cultural values and are delivered in our local languages will be better received.’ (P9, Female, 30 years, Durban-Chatsworth)

Other participants suggested using cultural expressions such as songs and dances to enhance the appeal of health campaigns. Participant 1 (P1) below added that:

‘Including cultural songs and dances in the campaign, events could make these gatherings more appealing and culturally relevant.’ (P1, Male, 32 years, PMB-Imbali)

Many pointed out the need for educational programmes that specifically address cultural myths surrounding health. Another suggestion focused on the need for health workers to receive training on cultural sensitivity. Participant 2 (P2) and P5 shared their suggestions as follows:

‘We need workshops that address the myths and facts about health in the context of our cultural beliefs.’ (P2, Female, 32 years, PMB-Imbali)

‘Training for health workers on cultural sensitivity could help them interact better with our community.’ (P5, Female, 26 years, PMB-Sobantu)

Such training ensures that health workers are equipped to deal respectfully and effectively with cultural nuances, which can enhance the effectiveness of health interventions.

The community members recognise the need for health interventions to be culturally congruent and sensitive to the traditions and beliefs of the community. Integrating these elements into health programmes is seen as essential for improving their effectiveness and acceptance within the community.

#### Subtheme 4.3: The importance of feedback

The participants’ perceptions of giving feedback reflect a solid and clear consensus on the importance of having accessible and diverse channels through which community members can express their views and influence the direction and operation of health programmes. They emphasise the need for these, to ensure that health initiatives remain responsive, transparent and adaptable to the community’s evolving needs and concerns. According to most participants, regular feedback sessions as a way to keep such intervention strategies aligned with community needs were suggested. These allow for ongoing adjustments to health interventions, ensuring that they remain effective and responsive to the needs of the community:

‘We should have regular meetings where we can talk about what’s working in the health programmes and what’s not.’ (P4, Female, 26 years, PMB-Imbali)

Suggestions such as using structured surveys to gather feedback systematically were mentioned. They stressed that surveys provide a quantifiable and structured way to collect feedback, which can be critical for evaluating and improving the outcomes of such health interventions. Participant 3 (P3) shared her thought that:

‘Maybe they could use surveys after each session to collect our opinions and suggestions.’ (P3, Female, 39 years, PMB-Imbali)

Younger participants suggested the use of technology to facilitate feedback. They indicated that online platforms could enhance accessibility for feedback, particularly among younger or more technologically inclined community members:

‘An online platform for feedback would help those of us who are tech-savvy to share our views easily.’ (P9, Female, 30 years, Durban-Chatsworth)

Many also suggested the use of interactive feedback sessions. They said real-time feedback during interactive sessions can provide immediate insights and perceptions about the intervention strategy. Participant 1 (P1) elaborated that:

‘Interactive sessions where we can directly talk with health workers and give our feedback in real-time would be very useful.’ (P1, Male, 32 years, PMB-Imbali)

The responses from the participants demonstrate a strong desire among community members for effective and varied feedback mechanisms that allow them to influence and shape the health programmes affecting their lives.

## Discussion

Community member’s experiences of the volunteer-led lung cancer awareness strategy have been described as impactful, highlighting the reported change in their health behaviour. Community members’ understanding of the intervention as a strategy to enhance health knowledge on lung cancer signalled that they accepted and acknowledged the volunteers and their work in raising lung cancer awareness in their community. In our study, the volunteers were at the same level and performed similar duties to the CHWs. From the literature, CHWs bridged the gap between the healthcare system and the communities by delivering community services ranging from supportive care to health education and screening.^14^ The findings reported in this study are in line with those of a Kenyan study, which demonstrated that CHWs were well-received in the communities they served and had the capacity to promote awareness and positive health-seeking behaviours.^7^ From the participants’ responses, some actions such as health-seeking behaviour improved because of the volunteer-led intervention strategy promoting education and awareness of lung cancer. The responses highlighted the appreciation of the volunteers in promoting lung cancer awareness, as a lack of knowledge and preconceived fears regarding the healthcare system may delay health-seeking behaviour. A recent study on health-seeking behaviour trends found that key facilitators include improved health education, enhanced healthcare resources, reduced costs and a sound healthcare system.^15^ Furthermore, it was found that offering support within the social environment is essential.^15^

The participants’ responses indicated mixed feelings about the delivery methods of the volunteer-led lung cancer awareness and mobilisation intervention. While some appreciated the personal touch of home visits, others suggested that group sessions or community events might reach a broader audience more effectively without compromising safety. The use of community gatherings, churches and so forth, is supported by Rachlis and colleagues, who stated that it ensured a higher number of community members reached for health awareness, compared to one-on-one individual health education sessions.^7^ However, we would argue that the approach should depend on the purpose of the awareness and the type of disease and/or condition it addresses. For example, for a condition that has been in the public’s attention for a long time, public gatherings may be the best approach, as demonstrated above. For a novel condition or a condition that not much is known about, perhaps a combination of approaches may be the best approach. Our study results indicate a preference for a combination of approaches, with a noticeable affinity for community-centred gatherings. Community members felt that collective engagement allowed for a more dynamic exchange of information and the opportunity to address questions and concerns. One would argue that this may not be an effective strategy for introverted personalities, as they may not be open and engaging in such setups.^16^ Further research is essential to fully understand the effectiveness of health awareness initiatives in public gatherings. The utilisation of health clinics was also preferred, indicating a greater recognition of immediate healthcare and ease of access.

Nonetheless, the value of door-to-door outreach was also recognised, especially for reaching those less likely to attend public meetings. The individual approach was particularly effective among older community members facing mobility challenges. Visiting people in their homes for health education was reported to be more personalised, which some community members appreciated. Door-to-door health education was recommended by Abbas and colleagues as an educational method; however, ensuring adequate resources for the CHWs was suggested, to ensure the sustainability of this type of educational method.^17^ Although community members preferred door-to-door, the risk and safety of community members were a concern. Letting an unfamiliar person into your home can be risky. This contributed to some community members’ reluctance to welcome volunteers. An investigative report from Soweto highlighted frequent violent incidents and mugging risks faced by CHWs during door-to-door visits in informal settlements, underlining the occupational danger of entering unknown households in high-crime areas.^18^ The community member’s responses validate the significance of multiple educational approaches and emphasise the need for strategic integration of these methods to maximise the effectiveness of health awareness campaigns in diverse groups.

Culture-sensitive health communication is essential in ensuring the inclusion of individuals from different backgrounds.^19^ Understanding the challenges and opportunities in delivering health education in culturally diverse settings is observed in the user’s responses as they suggested that it would be beneficial for future programmes to incorporate an understanding of cultural differences when planning community interventions. This approach can enhance effectiveness and ensure that the needs of diverse communities are met.^20^ Culture-sensitive health communication can ensure the reduction of gaps in health outcomes by making messages equally understandable, meaningful and effective.^19^ Cultural integration was perceived to respect and incorporate cultural beliefs while educating the community about the benefits of modern medical advancements. These include traditional versus alternative or modern medicine used to treat diseases such as cancer, as well as traditional beliefs and gender, race, ethnicity and language differences. Therefore, encouraging the development of strategies that respect and include local cultural contexts is vital. In addition, addressing myths within the cultural framework can help clarify misconceptions and improve health literacy in a culturally sensitive manner.^21^ Engaging the community through more involvement of community members and community leaders was a highlight for community members. In doing so, encouraging feedback to gain insight into the effectiveness of the interventions and the need for further improvements.

### Strengths and limitations

The study utilised qualitative research methods, enabling a deeper understanding of the participants’ experiences with the intervention strategy. Through FGDs, it fostered an in-depth exploration of community members’ experiences as recipients of the intervention aimed at raising lung cancer awareness and mobilising efforts at the community level. This allowed the researcher to explore complex issues and gain rich, contextual insight into their behaviour and experiences. The experiences articulated by the participants would have been overlooked using the quantitative research methodologies. Another notable strength of this study is that the study provides a unique user’s perspective on the lung cancer awareness interventions that are likely to be acceptable at the community level.

This study had limitations, including the small sample size of only 13 participants, which raises concerns about the rigour of the sampling process and the representativeness of the selected participants. The sample contained very few male participants (2 participant out of 13 participants), which may also have affected the study’s findings.

Because of limited resources, the MLCCP study were only limited to selected areas in the province, which were largely urban settings. This selection highlights concerns regarding the transferability of the results beyond the settings represented in the study. Therefore, there is a need to conduct a study that will include rural settings to get a more comprehensive outlook of how these programmes can be implemented in real world settings. However, there are valuable lessons to be learned from this study.

## Conclusion

Overall, the lung cancer awareness and mobilisation intervention strategy were perceived by users as an effective measure to increase community health education about health issues such as lung cancer. Limited awareness may pose a challenge to health-seeking behaviour and decreased health challenges. Community member’s positive feedback should encourage further intervention strategies focusing on community health awareness. Community health workers are well received by community members as their roles are better understood. Health policy on lung cancer community awareness and mobilisation needs to be strengthened to address the issues raised in this study such as cultural integration and enhanced community involvement.
